# Providing online weight management in Primary Care: a mixed methods process evaluation of healthcare practitioners’ experiences of using and supporting patients using POWeR+

**DOI:** 10.1186/s13012-017-0596-6

**Published:** 2017-05-25

**Authors:** Emily Smith, Katherine Bradbury, Lisa Scott, Mary Steele, Paul Little, Lucy Yardley

**Affiliations:** 1 University Hospital Southampton NHS Foundation Trust, Southampton, SO16 6YD UK; 2 Health Psychology, University of Southampton, Highfield, Southampton, SO17 1BJ UK; 3 Primary Medical Care, University of Southampton, Southampton, UK

**Keywords:** Obesity, Mixed methods, Process analysis, Healthcare practitioners, Weight loss, Digital intervention, E-health

## Abstract

**Background:**

An online weight management intervention (POWeR+) combined with a small amount of primary care healthcare practitioner support is effective in helping patients to lose weight, but little is known about how practitioners interact with the POWeR+ intervention or their experiences of providing support for patients using POWeR+. The aim of this study was to explore practitioners’ usage of POWeR+ and their experiences of providing support to patients using POWeR+.

**Methods:**

Set within a randomised controlled trial of POWeR+, practitioners’ usage of POWeR+ was automatically captured and a qualitative process analysis was conducted employing semi-structured telephone interviews with practitioners who provided support to patients using POWeR+. The usage analysis captured how 54 practitioners used the POWeR+ intervention. Thirteen telephone interviews explored practitioners’ experiences of using POWeR+ and providing patients with face-to-face or remote (email and telephone) support. Interview data were analysed using inductive thematic analysis.

**Results:**

Usage analysis indicated that almost all practitioners engaged with POWeR+. Pages which displayed patients’ progress and allowed practitioners to email patients were used the most. Practitioners found POWeR+ straightforward and easy to use. Some practitioners preferred providing support face-to-face, which they enjoyed more than remote support. A small number of nurses found providing non-directive support using the CARe approach (Congratulate, Ask, Remind) challenging, feeling it was the opposite of their normal approach. POWeR+ enabled practitioners to raise the topic of weight loss with patients, and POWeR+ was viewed as a superior alternative to existing weight management support which was limited in most practices. Still some practitioners found it difficult to fit providing support into their busy schedules.

**Conclusions:**

Overall, practitioners engaged well with POWeR+ and perceived providing patients with support whilst using POWeR+ as acceptable and feasible. CARe provides a potentially useful model for how practitioners can combine human and digital support in a cost-effective way, which could be useful for the management of other conditions. Some potential barriers to implementation were identified, which allowed modification of POWeR+. The findings suggest that implementing this cost-effective online weight management intervention in Primary Care would be feasible and acceptable to practitioners.

**Trial registration:**

ClinicalTrial.gov, ISRCTN21244703

## Background

The prevalence of obesity is continuing to rise globally, predisposing individuals to a wide range of health conditions [1, 2]. By 2010, 3.4 million deaths and 3.8% of all disability-adjusted life years per year were caused by overweight or obesity [3]. Primary Care would like to provide help to obese patients, but lacks the resources needed to treat the growing number of obese adults, and practitioners frequently lack the necessary training to provide the behavioural counselling which could help people to lose weight [4]. Digital interventions (DIs) may provide a solution as they can provide behavioural weight loss support at relatively low cost without the need for practitioners to learn behavioural counselling techniques [5]. The downside of DIs is that engagement may be poor [6], but the addition of a brief amount of human support can improve engagement and outcomes [7].

It is not yet established what amount or type of support (face-to-face or remote involving telephone or email) might be optimal for supporting DIs [8]. We recently conducted a large clinical trial which tested the effectiveness of a digital weight management intervention called POWeR+, accompanied by brief face-to-face or remote (telephone or email) support from a healthcare practitioner (HCP). HCPs provided support using the CARe approach (Congratulate, Ask, Remind; see the ‘Methods’ section for an overview of CARe) [9]. Results showed that both groups were effective, with no significant difference in weight loss between those receiving face-to-face or remote support; 29% of the face-to-face and 32% of the remote support group had a clinically significant weight loss of 5% or more of their body weight by 12 months.

Evidence indicates that there are a number of barriers to successfully implementing interventions like POWeR+ in practice. Barriers can be contextual (e.g. financial incentivisation of only certain behaviours), organisational (e.g. limited resources) or professional (e.g. perceiving the practitioner role as purely biomedical) [10, 11]. Many interventions which aim to change HCP behaviour, in order to change patient health outcomes, have only modest effects, or no effect at all [10, 12, 13]. It is therefore vital that researchers explore how new interventions are used and experienced by HCPs to identify potential barriers to their wider implementation in practice. Process evaluations are recommended in national guidance [14] and can help to identify how healthcare is implemented, the likely mechanisms through which an intervention produces an effect and important factors within the healthcare context which might influence the delivery of an intervention. Review evidence has highlighted that trials often fail to carry out process analyses, which can lead to problems in understanding how interventions might produce their effects and why they might (or might not) be successful in practice [15]. The current study presents a quantitative process evaluation of HCPs’ usage of POWeR+ and a qualitative process evaluation of HCPs’ perceptions of providing support to patients using POWeR+. We were interested in understanding how practitioners engaged with POWeR+; our research questions for our quantitative usage analysis were ‘What aspects of POWeR+ were used by HCPs?’ and ‘How many times did they use each aspect of POWeR+?’ Our research questions for our qualitative process analysis asked ‘How did HCPs experience using POWeR+ and providing support to patients who were using POWeR+?’ and ‘What factors might be potential barriers to HCPs carrying out the POWeR+ intervention as intended?’ This process evaluation could tell us more about the feasibility of implementing this intervention in Primary Care and could highlight potential barriers to implementation and modifications necessary for successful implementation.

## Methods

### Study design

We took a mixed methods approach with concurrent data collection underpinned by a pragmatic perspective [16]. The qualitative process analysis used semi-structured interviews to gain a rich, in-depth understanding of HCPs experiences of using POWeR+ and supporting patients using POWeR+ and potential barriers to carrying out the intervention as intended. The quantitative usage analysis explored HCPs engagement with the POWeR+ website. We carried out a composite analysis, whereby both the quantitative and qualitative components were conducted as complete components and the data analysed separately and then integrated for discussion [16]. This approach ensures that the unique characteristics of each method are not lost. Whilst data from each of these methods was seen as complementary, the qualitative data was given more prominence in the analysis since it provides richer, more detailed insights into how the POWeR+ support was implemented and experienced as well as how feasible POWeR+ might be to implement in practice.

NHS ethics and RGO approvals were awarded, and the study was registered with Current Controlled Trials (number ISRCTN21244703).

### POWeR+

POWeR+ consists of two interfaces, one for patients and one for the HCPs providing support to both intervention groups. The patient website is explained more fully elsewhere [17–19] but in brief provides patients with a choice of a low calorie or low carbohydrate eating plan and a physical activity component. POWeR+ aims to help patients develop self-regulation skills by promoting weekly weighing, eating and physical activity goal reviews, as well as online sessions based on cognitive-behavioural techniques.

The HCP interface provides brief information about POWeR+ and how to provide support to patients (this information was also given on paper in HCP study files). The website also allowed HCPs to view patients’ recorded weekly weight and goals and send patients support emails. Patients in the face-to-face group were offered three scheduled (and four optional) face-to-face support sessions with phone or email contact used if patients did not attend in person. The remote support group had three scheduled phone or email contacts and two optional phone or email contacts. The optional support in both groups was triggered by weight gain or patient request.

### HCP support

In the first version of POWeR [7], we gave HCPs access to detailed information about the patient website hoping that practitioners would therefore be able to give advice that was consistent with POWeR. However, HCPs reported that they lacked the time to look at this information, and usage analysis revealed that very few had looked at these support pages. Taking a Person-Based Approach [19], we redesigned the HCP support for POWeR+ based on practitioners’ feedback. In POWeR+, practitioners were asked not to give advice to patients, instead all advice came from POWeR+ and the practitioners simply provided a supportive relationship to promote adherence.

We developed the CARe approach (Congratulate, Ask, Remind) to facilitate a non-directive supportive relationship which would be easy to deliver and would fit with practitioners’ busy schedules. In addition to being developed using a Person-Based Approach, the CARe approach is also theory based, grounded in Self-Determination Theory (SDT; [20]). CARe aims to provide an autonomy supportive relationship which can raise patients’ autonomous motivation for behaviour change by promoting feelings of autonomy, competence (feeling effective) and relatedness (feeling understood and cared for by others) [21]. Autonomy supportive relationships with HCPs predict better weight loss outcomes [21, 22] as well as predicting outcomes in a range of other health conditions [22]. The theorised mechanisms of the CARe approach are explained in Table 1.

**Table 1:** The CARe approach—Congratulate, Ask, Remind

Guidance given to practitioners about CARe	Theoretical basis
Congratulate the patient on any use of POWeR programme: • You can congratulate them on any weight lost, achieving goals, setting goals, or just logging in! • Even if they have not logged in or used POWeR, remember to be positive. They may have been ill or on holiday. • If they have not used the programme yet, congratulate them on consenting to take part in the study, which shows they are interested in managing their weight—just logging into the programme is a step in the right direction	Praise was focused on the process of behaviour change (e.g. ‘great job on sticking to your goals’, or ‘well done on losing weight’), rather than focused on the person (e.g. ‘you’re great at losing weight’). Autonomous motivation and feelings of competence can be enhanced by process focused praise [31, 32]. Praise was designed to be informational (‘That’s great that you’ve logged on and had a look at POWeR+’), instead of controlling (‘Well done you’ve logged on to POWeR+, as you should’) which also supports autonomy [31, 33]. No pressure was put on participants who had not engaged with behaviour change, as avoiding pressure supports autonomy [21].
2. Ask the patient if they have any questions or concerns about making lifestyle changes, and then: • Ask the patient what solutions they would like to try—remember, the aim of POWeR is to encourage people to become their own health trainer, not to rely on others. • Direct patients to their tools’ section (for further information on a range of topics) • If they have not lost weight, ask if they have tried using a food diary for a few days to work out what foods or drinks to swap or cut down. You can show them how to work this out using a calorie counter (if on the low calorie plan).	Asking about potential barriers and exploring possible solutions with patients can build more autonomous motivation [33]. It could also help patients to feel understood and cared for, and so enhance relatedness. In this case, emphasis was put on discussing the patients’ (rather than HCP’s) ideas of possible solutions to challenges, to help build their feelings of competence and to help them to rely on themselves, rather than the HCP, for solutions. If patients were struggling to lose weight, then HCPs could suggest that patients self-monitored their dietary intake more closely for a short period of time, to understand where they might need to make changes, to help build feelings of competence.
3. Remind the patient about future support from you. • You can explain to patients that you will be following their progress online and that they can email you for advice about using POWeR if they want to.	HCP monitoring patients’ progress online could potentially enhance external, rather than autonomous, motivation. However, minimising pressure can help support [21], likely negating some of this effect. This was achieved by mentioning monitoring only in the context of telling patients that they could access more support if they wanted to. Providing choice (in this case about whether and when to receive additional support) also helps to support autonomy [21]. Offering the opportunity for additional support might also enhance feelings of relatedness [34].

### Quantitative usage analysis

HCPs usage of the POWeR+ website was automatically collected by the Lifeguide software (https://www.lifeguideonline.org/), which provides an objective measure of how many practitioners viewed each page and how many times. This data was downloaded at the end of the 12-month trial. We examined the website usage of all HCPs and also compared the usage of those who had and had not taken part in our qualitative process study to explore any differences between these groups.

We expected that webpages which enabled HCPs to look at patients’ progress and provide email support to be used by all practitioners (pages 9–12; see Table 2 for an overview of webpage content). However, we were not sure how many practitioners might access the other parts of the HCP website, since HCPs might remember the topics covered from their initial training or by looking at their site files.

**Table 2:** POWeR+ HCP usage analysis

Description of pages in POWeR+ for HCPs	Users who viewed the page at least once (*N*)	Total page views (*N*)
Page 1: Homepage Includes a welcome message and a brief description of POWeR+	54	2588
Page 2: Why use POWeR+? Information about the POWeR+ intervention	18	27
Page 3: The POWeR+ philosophy Information about the key principles of POWeR+	18	23
Page 4: Meet the POWeR+ team Information about the team who created POWeR+	16	21
Page 5: POWeR+ and diabetes Additional considerations for diabetic participants	25	39
Page 6: Differences in group support Tells the HCP about the different study groups and what each should receive	38	80
Page 7: HCP support schedule diagram	40	104
Page 8: How to provide support to those using POWeR + Tells the HCP about the CARe approach	41	112
Page 9: Patient summaries The HCP can click on a patient to see their goals and weight loss	54	5622
Page 10: Page showing individual patient’s current goals and record of weekly weights	53	5143
Page 11: Sending support emails to patients The HCP can send the patient an email from here using template emails that they could modify or replace with their own text	52	1585
Page 12: Email confirmation page This confirms to practitioners that their email has been sent and gives the HCP the option to send another email	45	992

### Qualitative process analysis

#### Procedure

The 54 HCPs who supported POWeR+ were nurses (*N* = 53) or healthcare assistants (*N* = 1); almost all were female (*N* = 53). All HCPs were sent a study invitation letter, information sheet and consent form. Nineteen female nurses expressed an interest in taking part, and 13 were interviewed by telephone. No new themes emerged with later interviews implying that saturation had been achieved [23]. Practitioners who chose not to participate noted that they did not have time.

The telephone interviews were conducted between April and June 2014, approximately 6 months after a practice had begun POWeR+ when the nurse support element of the intervention was completed. The interviews were conducted by LP, a health psychology MSc student who was given training in qualitative interviewing and analysis. LP had no prior relationship with any of the HCPs interviewed. HCPs who agreed to participate posted a signed consent form to the researcher prior to the interview.

Before each interview, LP explained that she was a postgraduate student with no previous involvement in the design or evaluation of POWeR+. The interview schedule was developed based on our feasibility trial and development work with HCPs and explored HCP experiences of providing support for POWeR+ participants, their experiences of using the POWeR+ website and, of the study procedures, examining barriers to engaging with POWeR+ and implementing the intervention as planned (see Appendix 1 for interview schedule).

#### Analysis

All interviews were audio recorded and lasted between 23–46 min. The recordings were transcribed verbatim and imported into NVivo 10 to allow systematic comparisons to be made across the data set. An inductive thematic analysis [24] was carried out, augmented with procedures from grounded theory [25]. Firstly, the researchers familiarised themselves with the data. The interviews were then coded, and a coding manual was created. This coding manual was continually updated to reflect the on-going analysis. Constant comparison was used to ensure that codes were being used consistently and reflected the data [25]. Codes which identified similar aspects of the data were clustered together into themes. Inter-rater agreement on all the final codes and themes was agreed with ES (a post-doc research fellow), LY (a health psychologist) and LP. Deviant case analysis was used to ensure that perspectives which diverged from dominant trends were not overlooked.

## Results

### Nurse support provided

The nurse support provided is reported fully in our trial paper [9] but is briefly summarised here. In the face-to-face group, a mean of 4.5 contacts were provided, consisting on average of 2.3 face-to-face, 2.13 emails and 1.8 phone contacts. In the remote support group, a mean of 4.35 contacts were provided, consisting on average of 3.13 emails and 1.62 phone calls. Eighteen patients also received a small amount of face-to-face support in this group.

### Quantitative usage findings

An overview of the usage analysis is provided in Table 2. The usage analysis revealed that all 54 HCPs used the POWeR+ website. All practitioners looked at the patient summaries (page 9) and nearly all looked at patients’ individual goals and weight loss progress (page 10). The very high amount of total visits to these pages indicates that practitioners were using them many times during the study to provide support to patients. Forty-five HCPs sent an email to patients during the study through POWeR+, with 992 emails sent in total (page 12). Most practitioners (*N* = 38–41) looked at pages which provided further information about how to provide support to participants (page 6–8). A smaller number of practitioners (*N* = 16–25) looked at the pages which provided information relating to the POWeR+ website, its philosophy and the team who created it, as well as additional information for participants with diabetes (pages 2–5).

An overview of the web-pages used by HCPs who took part in an interview is presented in Table 3. A notable finding was that one practitioner (HCP 9) who reported positive perceptions of using POWeR+ and providing support to patients during interview only actually viewed the homepage of POWeR+ and not the rest of the online content. Table 3 also shows the average (Mean) number of times each POWeR+ webpage was viewed by HCPs who did or did not participate in interviews. Most webpages were viewed a higher number of times by interviewees than non-interviewees, in particular the pages relating to patient summaries and sending patients emails (pages 9–12); this may have been because interviewees had more patients on average to provide support to (Mean = 18.2) compared to non-interviewees (Mean = 14.7).

**Table 3:** POWeR+ usage by HCP interviewee

HCP	Patients per practice	POWeR+ page views (*N*)											
		Page 1	Page 2	Page 3	Page 4	Page 5	Page 6	Page 7	Page 8	Page 9	Page 10	Page 11	Page 12
1	16	100	2	2	2	5	5	9	6	216	206	55	29
4	19	43	0	0	0	0	2	0	1	106	95	74	55
5	17	84	5	3	2	4	2	2	4	148	131	70	37
6	25	59	0	0	0	1	3	4	2	153	144	17	7
7	27	88	0	0	0	0	0	1	0	137	128	19	9
8	14	76	0	1	0	0	0	1	0	108	92	10	2
9	10	1	0	0	0	0	0	0	0	0	0	0	0
12	8	23	0	0	0	1	0	2	6	68	65	18	12
13	23	65	0	0	0	3	1	0	1	187	181	28	16
15	23	104	0	0	0	0	2	3	3	180	171	37	22
17	20	89	0	0	0	0	2	0	1	375	360	93	77
18	19	36	0	0	0	0	0	0	0	71	67	30	23
19	16	47	1	2	2	1	1	6	6	167	157	48	31
Mean : interviewees	*18.2*	*62.7*	*0.6*	*0.6*	*0.5*	*1.2*	*1.4*	*2.2*	*2.3*	*147.4*	*138.2*	*38.4*	*24.6*
Mean : non interviewees	*14.7*	*43.2*	*0.5*	*0.4*	*0.4*	*0.6*	*1.5*	*1.9*	*2.0*	*90.4*	*81.6*	*26.5*	*16.4*

**Fig. 1:**
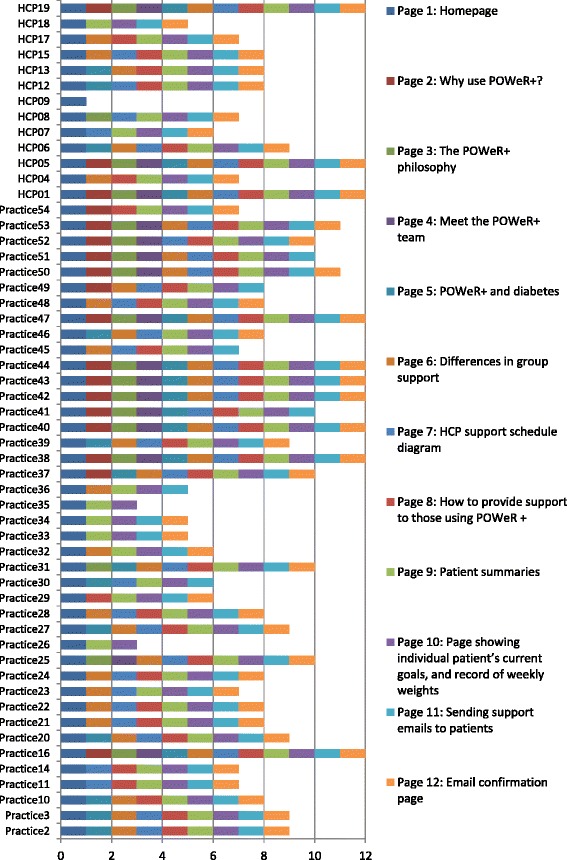
POWeR+ pages visited by interviewees (HCP) and non-interviewees (practice). This figure shows the POWeR+ pages visited by interviewees (labelled as HCPs) and non-interviewees (labelled as practices)

Figure 1 shows the range of POWeR+ webpages which each HCP viewed. As we expected, the majority of practitioners viewed the pages which were most vital to providing support (pages 9–12). Specifically, 53 practitioners viewed the pages relating to viewing patients progress (pages 9–10), 51 practitioners viewed the facility to send patients an email (page 11), and 45 practitioners sent patients an email through this facility (page 12). This figure shows that only 3 practitioners were low users of POWeR+, with 2 only viewing the parts of POWeR which were most vital to providing support (the patient summaries, pages 9 and 10) in addition to the homepage and 1 practitioner viewing only the homepage (HCP 9).

### Qualitative findings

Four themes were identified: ‘HCPs’ perceptions and use of POWeR+’, ‘Supporting patients in their use of POWeR’, ‘The impact of POWeR+’ and ‘Comparisons to existing weight management services’.

### HCPs’ perceptions and use of POWeR+

HCPs reported finding the POWeR+ website straightforward and easy to use.

I found it quite easy to sort of navigate around and you know, dip in and out of. Yeah, no I found it quite, quite user friendly certainly. (HCP-15)

Practitioners’ views about the content of POWeR+ varied; some were very positive, describing the website as ‘Very comprehensive’ (*HCP-01*) while others felt it was adequate: ‘Basic I would think but quite satisfactory’ (*HCP-12*)*.*

One feature of POWeR+ that HCPs found particularly valuable was the email prompts, which reminded HCPs of key actions that they needed to take like contacting patients for support appointments. Practitioners viewed these as very helpful in their busy work environments.

It’s useful to have the emails just to flag up and I used to leave them in my inbox just as a reminder until the patient you know had been dealt with (HCP-15)

A few practitioners voiced frustration that they could not see the information given to patients on the POWeR+ website. They felt that seeing this information would have allowed them to understand more fully what patients were referring to during consultations. However, some did acknowledge that their time was very limited, and therefore, it might have been challenging to view this additional information.

I don’t know what they’re reading … they don’t understand that and I find that difficult. It would be easier to talk to them about it if we knew what they were looking at. (HCP-08)

There is no time to sort of browse, so the things I’m saying to you that may well have been on there and I didn’t actually get round to looking at them. (HCP-05)

A few practitioners found it challenging to avoid giving patients specific advice, as practitioners were used to directing patients to what they thought was the best solution and worried that patients might expect this, rather than the non-directive support used in the CARe approach.

Just to give general advice and nothing specific. So, that was hard, yeah, compared to my job where you’re doing, yeah, the opposite all the time. So, that was a little bit difficult and I think perhaps a little frustrating for some of the patients, that they didn’t get more out of me. (HCP-19)

I’m not allowed to sort of, what’s the word, guide them too much…so all I can sort of do is point them in the direction of the website, you know, and it, sometimes you just want to say something and you can’t, so I find that quite difficult. (HCP-07)

Some HCPs suggested that it would have been good for the patients to have been able to leave comments for the practitioner to see.

All I got to see was their goals and any weights they've logged in. […] if they’d made any comments about how they’d had a particularly good week or bad week or anything like that, might’ve been quite interesting, just to have sort of been able to see that as well. (HCP-18)

### Supporting patients in their use of POWeR+

HCPs supported patients in both the face-to-face and remote support groups.

HCPs enjoyed providing face-to-face support, and some expressed a preference for this rather than providing remote support.

Ideally I’d like everybody to be in a face-to-face group really. That was the most satisfying for us … we’re not so used to communicating online with people, and I feel you get far more out of a conversation if you’re actually looking at someone. (HCP-05)

Some HCPs felt they got the best response from participants in the face-to-face support group.

Those that were in the remote group I felt really had to have a lot more willpower just to keep going really, and with faceless emails coming, but those that were in the face-to-face groups did really well. (HCP-05)

However, some practitioners reported struggling to get some patients to attend appointments.

Views about remote support were mixed; on the one hand, some nurses found face-to-face support more enjoyable, but on the other hand, they understood that remote support might be more practical for some patients.

I suppose the face-to-face is nicer from the relationship you get with the patients, but on the other hand the email can be perfectly efficient and a lot of people lead very busy lives, they don’t want to be coming in. (HCP-06)

It appeared that some HCPs did not view remote support as support at all; one nurse noted that patients in the remote support group would ‘probably [be] disappointed that they didn't get any support’ (*HCP-12*)*.*

Some HCPs reported that remote support was difficult as patients were not always available by phone. Equally, supportive emails through POWeR+ were outgoing only, meaning that nurses did not know whether the emails were being read and whether their support was helping. This concern may explain why not all practitioners provided email support through POWeR+.

I didn’t go about phoning because that was just too difficult ‘cos when you’re in the surgery they might not be there … so it’s actually easier for people to email them. (HCP-08)

I wasn’t sure whether the person had got my email maybe, or if there was no feedback, so if they hadn’t logged on or something, then I had no means of knowing if they were interested anymore. (HCP-13)

### The impact of POWeR

Participating in the POWeR+ trial was reported to impact on practices in a variety of ways. A small number of nurses reported that POWeR+ raised their awareness of the damaging impacts of obesity on physical and mental health, as well as how much improvement weight loss could make to health conditions.

We’ve had one lady who’s gone from being hypertensive on two therapies to now on no medication at all…that was a huge sort of wake up call to me actually how much difference you could make to someone’s life. (HC-09)

The availability of POWeR+ was occasionally viewed as a useful opportunity to help other nurses and GPs to broach the topic of being overweight with patients.

[Practice staff] were quite keen to put people my way and I think sort of GPs and nurses found it a useful place to send people because weight loss is quite a difficult subject sometimes, it can be um can be quite hard to either broach or work through so I think it was useful that they had something that was on offer. (HCP-04)

There were mixed views on the impact providing support had on practitioners’ time. HCPs from research active practices or with flexible working practices felt that supporting patients with POWeR+ had only a little impact on practice time. For HCPs who did not have time allocated specifically to research, managing the logistics of providing support for patients could be challenging. Booking time out in their diaries to provide support for POWeR+ patients was reported as a good solution.

For the first few weeks I did feel I was possibly a little bit behind…Obviously, seeing the patients is fine 'cause they were booked in to my clinic so I had that time, but it was the follow-up over phone calls or emails. So once I realised that and I could book the time then I managed to sort of keep on top of it much more. (HCP-18)

### Comparisons to existing weight management services

HCP’s drew comparisons between POWeR+ and what was on offer to patients through their practice or available to patients externally in the form of slimming clubs and groups. Many practitioners discussed how their practice offered a fairly limited service for people who wanted to lose weight. POWeR+ was therefore seen as a useful alternative.

I have to say that we don’t give an awful lot. When patients come in we do their height, weight, their body mass index, you know, if they're on a chronic disease register or if they come in for an NHS health check then we do tend to address their height, their weight, their body mass index. But there isn’t anything really formal that we give… We need a standard response, and I think having the POWeR tools means we’re all singing from the same hymn sheet. (HCP-09)

Some HCPs did report that ordinarily they would allow patients to come into the practice to weigh themselves but that formal support was not available. One HCP mentioned that routine weighing had stopped for cost reasons:

We did offer patients regular sort of weigh-ins so to speak. If they wanted to they could come back every couple of weeks or a month but the practice has since then decided that we wouldn’t be offering this service because it was, this will sound terrible, a waste of money. (HCP-05)

Others reported that they could refer patients to external weight management groups. POWeR+ was viewed as having similar or better content than commercial products, but without cost to the patient. POWeR+ was also seen as a useful option for people who did not want to attend group meetings.

One lady I had she wanted to do Weight Watchers and Slimming World and all this but she couldn’t afford to whereas being on the programme, she’s got probably more information in a similar format, for free. (HCP-01)

Well with Weightwatchers I think you can do online, but you have to go to a group first, so it, for people who really can’t face going to a group session it gives them an alternative which is endorsed by their doctor. (HCP-06).

## Discussion

The current study explored HCPs’ usage of POWeR+ and their experiences of supporting patients who were using POWeR+. The usage analysis indicated that all practitioners used POWeR+. The most used parts of POWeR+ were the summaries of patients’ progress and a page where practitioners could send patients emails. It is perhaps unsurprising that these parts of the website were used the most since they directly related to what practitioners needed in order to provide support to patients. It was surprising that 9 out of 54 practitioners did not send patients any emails through POWeR+, since this was quick to do. However, we cannot rule out that practitioners were sending emails to patients through their personal emails instead. Alternatively, it may be that these practitioners did not send emails as they did not feel comfortable using email to provide support, as highlighted by a few practitioners in our qualitative process analysis.

When we compared the usage data of those who participated in the qualitative study with those who did not, interviewees were more frequent in their use of the POWeR+ website but did not appear any more likely to view all of the POWeR+ webpages. It was surprising to find that one interviewee (HCP 9) who talked positively about supporting patients and about the POWeR+ content had in fact not used the POWeR+ website beyond viewing the homepage. It could be that in this case, they used their own practice email and the paper support file which contained much of the website documents to provide support.

Within the qualitative process analysis, HCPs reported finding POWeR+ easy to use and its content acceptable. They generally enjoyed supporting patients using POWeR+ and often perceived POWeR+ as superior to the weight loss services that were available in their practices. HCPs also highlighted a number of challenges that they faced in providing support for POWeR+, which might be barriers to its future implementation. Table 4 outlines each of these challenges and how they will be addressed in the version of POWeR+ that will be disseminated for future use. Where appropriate, we have triangulated the practitioner feedback with our main trial findings [9] and qualitative patient process interviews [manuscript in preparation: Smith, Mowbary, Bradbury, Little, Yardley; Patients’ perceptions of POWeR+: A qualitative interview study] to inform our response.

**Table 4:** Plans for addressing the challenges faced by practitioners

Challenges faced by practitioners	Plans for addressing challenges
A few practitioners wanted to see the information which was given to patients on the POWeR+ website.	This information will be made available to HCPs. Since many practitioners have limited time, viewing these patient pages will be considered optional. Practitioners will not need to know the content of the patient website in order to provide support.
Some practitioners found it challenging not to give specific advice as they were concerned that some patients might expect this.	In future, our online training for POWeR+ will present practitioners the evidence that the CARE approach with no advice from the practitioner is effective (based on our trial evidence; [9]), and that patients like it (data triangulated from patient interviews; [Manuscript in preparation: Smith, Mowbary, Bradbury, Little, Yardley. Patients’ perceptions of POWeR+: A qualitative interview study]), to persuade practitioners that they are not doing patients a disservice by not providing advice. We took this approach in another of our interventions and found that it reduced HCP concerns about not providing advice [Manuscript submitted: Bradbury, Morton, Band, May, McManus, Little, Yardley, understanding how primary care practitioners perceive an online intervention for the management of hypertension].
Practitioners felt it would be useful for patients to be able to leave comments for them with their weight data, to give them further information about how the patient was getting on. This contextual information might help practitioners to provide more personalised support.	Patients also commented that they would have liked to leave comments for the practitioner when entering their weight into POWeR+ [Manuscript in preparation: Smith, Mowbary, Bradbury, Little, Yardley. Patients’ perceptions of POWeR+: A qualitative interview study]. This facility will therefore be added to POWeR+.
Some practitioners had a preference for providing face-to-face rather than remote support, a few felt that this was more valuable to patients.	In our online HCP training for POWeR+, we will reassure practitioners about the value of remote support by showing them the evidence that remote support is as effective as face-to-face support (as shown in our trial; [9]) and that remote support is also acceptable to patients and preferred by some patients (data triangulated from patient interviews; [Manuscript in preparation: Smith, Mowbary, Bradbury, Little, Yardley. Patients’ perceptions of POWeR+: A qualitative interview study]).
Some practitioners found it difficult to fit the POWeR support into their busy schedules.	We will suggest to practitioners that they book time in their diaries for providing support, as other nurses found this to be an effective strategy for enabling support to fit into their schedule. As remote support is likely to take less time and is as effective as face-to-face support, then we can recommend that practitioners provide most support remotely.

The findings identified within our inductive analysis also map well onto constructs from Normalisation Process Theory (NPT), which describes factors necessary for successful implementation in practice [26, 27]. Here, we map our findings onto NPT to allow consideration of how well POWeR+ might implement in practice. Our qualitative findings suggested that the POWeR+ intervention made sense to HCPs (coherence). There was also evidence that practitioners cognitively participated in the POWeR+ intervention (i.e. there was ‘buy in’ to the intervention), as they viewed providing support as a useful thing for them to do. However, delivering support remotely was a new approach for a few practitioners, perceived as less valuable than their usual face-to-face approach, and some practitioners did not provide email support through POWeR+. Additionally, a few practitioners wanted to give advice to patients, rather than only provide non-directive support using CARe, which suggested they might not have fully bought into this procedure.

There was evidence of collective action, as support logs indicated that all practitioners provided support to patients. HCPs noted that the emails from POWeR+ facilitated this action, which they appreciated. However, there were also some threats to collective action, most prominently a lack of time, which is perhaps unsurprising, given the current pressures in Primary Care. In the POWeR+, trial remote support (consisting on average of 1.6 phone calls and 3.3 emails) had the same effect as face-to-face support [9]; therefore, providing remote support might be a more feasible option for HCPs with very limited time. However, some practitioners reported a preference for providing face-to-face support. We hope to persuade practitioners of the value of remote support in the version of POWeR+ that we will implement in practice, by showing them that remote support was just as effective and acceptable to patients as face-to-face support [9], but further study of how practitioners respond to this evidence at implementation will be useful.

Practitioners were positive in their appraisal of the effects of POWeR+ (reflexive monitoring), indicating that the intervention had improved their understanding of the effects of obesity and weight loss and had proved a useful tool for treating obese patients for themselves and colleagues, which was viewed as equal to or better than commercial products. Reflecting on our findings using NPT, we can identify several potential threats to the normalisation of POWeR+ in practice, but as we have been able to address many of these before disseminating POWeR+, we hope to have reduced potential problems with implementation. Further exploration of how POWeR+ implements in practice will be useful to ascertain whether our modifications were successful and if more are needed to ensure optimal implementation.

We were unable to find any other studies which have explored HCPs usage of an online intervention. Additionally, very few studies have previously looked at HCPs’ perceptions of providing support for online weight loss interventions. Two focus group studies have explored physicians’ perceptions of providing support for patients using an online intervention [28, 29]. However, in both these studies, the physicians provided infrequent support to patients (every 3–6 months), whilst a coach provided patients with weekly intensive support. Coaches were not interviewed in these studies, meaning that only the perceptions of physicians who provided relatively sparse support were explored. Still, these studies usefully show that some physicians perceive that they lack the skills to support patients losing weight [29] and that others use dubious motivational strategies, such as expressing frustration to patients who are not making progress [28]. These findings suggest that a more structured approach to providing support, such as the CARe approach, may be a useful tool for practitioners wanting to support patients using online weight loss interventions. The wider literature examining healthcare practitioners’ experiences of providing support for an online intervention in chronic health conditions tends to be of slightly lower quality, due to very small samples of practitioners. This literature suggests that HCPs see a lack of time as a key challenge to providing support [29, 30], which is consistent with the findings of our study and represents a challenge to implementing DIs into healthcare settings. One solution may be to provide patient supprot remotely, mainly through emails which are quick to send. Our trial indicates that brief phone and email support achieves similar weight loss outcomes but is more cost effective than providing face-to-face support [9].

### Strengths and limitations

This is the first study to compare HCPs’ experiences of providing face-to-face and remote support for an online intervention. A key strength of this study was that HCPs’ interview data could be triangulated with their usage data, as well as data from patient interviews, patients’ engagement with POWeR+ and weight loss outcomes from the POWeR+ trial, providing a fuller understanding of the data. We used a composite analysis approach, conducting our quantitative and qualitative process evaluations as separate studies, which allowed us to maintain the unique features of both datasets [12]. However, it is possible that looking at an interviewee’s usage data during their interview might have enabled exploration of why some aspects of POWeR+ were used less than others. Whilst only 19 HCPs agreed to be interviewed, and 13 were interviewed, no new themes emerged from later interviews. It is possible that the interviewees might differ from the non-interviewees in some way. Indeed, our usage analysis indicates that interviewees visited most POWeR+ webpages more times than non-interviewees, which could indicate they might have more positive views of the intervention. However, the interviewee who used very little of POWeR+ (HCP 9) had positive perceptions of POWeR+ and of providing support, suggesting that lower usage does not necessarily indicate less positive perceptions of the intervention. This study focused on exploring potential barriers to implementation at the practitioner level by inductively exploring HCP’s experiences of using POWeR+ and supporting patients who were using POWeR+. We employed an inductive approach, rather than using a theory-based deductive approach based on theorised barriers to implementation, as we wanted to elicit views and experiences without prompting conversation on known barriers in order to reduce the risk of influencing the barriers discussed. Whilst organisational level barriers were mentioned by interviewees (e.g. lack of resources), there may be other barriers to implementation at a wider organisational level which were not captured. For example, elsewhere nurses have indicated that lack of payment through the Quality Outcomes Framework for providing self-management support is a barrier to providing self-management support to patients [11]. Further research exploring the perceptions of people who might influence the implementation of POWeR+ in practice, such as practice managers or Clinical Commissioning Groups, would be useful to look at wider barriers to implementation as we disseminate POWeR+.

## Conclusions

This study highlights a number of potential barriers to the successful implementation of a highly cost-effective weight-management intervention, POWeR+, and shows how we modified POWeR+ in order to overcome these barriers prior to implementing POWeR+ in practice. The current lack of resources within Primary Care means that weight-management interventions of this kind are desperately needed and POWeR+ is now being disseminated within Primary Care and Public Health. This study showed that HCPs generally engaged well with POWeR+ and found it feasible and acceptable to support patients using POWeR+. The modifications to POWeR+ that this study enabled should improve the intervention chances of normalising in practice. Further research would be useful to explore how well POWeR+ normalises in practice. DIs are rising in popularity, and additional human support is likely to maximise the effectiveness of these interventions. The CARe approach shows initial promise as a model to guide human support for digital weight loss interventions and may be a useful and cost-effective [9] model to guide human support in DIs in other conditions. It would be useful for future research to test the effectiveness, acceptability and feasibility of CARe as a method for providing human support for DIs in a range of health conditions.

## Appendix 1

### Interview schedule

1. Can you tell me about your overall experience using the healthcare professional website? (Probe around any potential problems or barriers to use)
2. What do you think of the POWeR+ website content? (Probe around what they used and any problems encountered)
3. Can you tell me about the study emails you received (check that emails were received)? What do you think of these emails? (Probe around any helpful/unhelpful aspects of the emails)
4. How does supporting patients who are using POWeR+ differ from your normal practice? Would you normally offer weight management advice? (If yes,) can you tell me how this works? How does POWeR+ compare?
5. What did you think of delivering the support appointments? Can you tell me all about your experiences of these? (Probe around any problems raised to explore barriers to supporting patients)
6. What do you think could be improved in the POWeR+ intervention? (Explore in relation to the *whole intervention*: both website and delivering support)
7. What do you think is good about the POWeR+ intervention? (Explore in relation to the *whole intervention*: both website and delivering support)
8. What do you think patients thought of the intervention?
9. What do you think the impact of this support was on patients?
10. What would you do differently if you were to repeat this intervention in practice? What would you keep the same?
11. What impact did taking part in POWeR+ overall have on:
  - The practice?
  - The patients?
  - You?
12. Is there anything else you would like to add that we haven’t yet discussed that could help us to design, develop or test similar web-based programmes in the future?

## Abbreviations

CARe: Congratulate, Ask, Remind
DI: Digital intervention
HCP: Healthcare practitioner
